# Supplementary material for: CUTie2: The Attack of the Cyclic Nucleotide Sensor Clones
Source: Front Mol Biosci. 2021 Mar 11;8:629773. doi: 10.3389/fmolb.2021.629773 (PMC7991088; doi:10.3389/fmolb.2021.629773)
Supplement: Supplementary file 1 [file datasheet1.pdf]

Supporting Material to:

CUTie2: The Attack of the cyclic nucleotide sensor clones

Florencia Klein[1,2], Florencia Sardi[3], Matías Machado[1], Claudia Ortega[4], Marcelo Comini[3], Sergio Pantano[1*]

[1] BioMolecular Simulation Group, Institut Pasteur de Montevideo, Montevideo, Uruguay. Mataojo 2020, CP 11400
[2] Graduate Program in Chemistry, Facultad de Química, Universidad de la República, Uruguay.
[3] Laboratory Redox Biology of Trypanosomes, Institut Pasteur de Montevideo, Montevideo, Uruguay. Mataojo 2020, CP 11400
[4] Recombinant Protein Unit, Institut Pasteur de Montevideo, Montevideo, Uruguay. Mataojo 2020, CP 11400

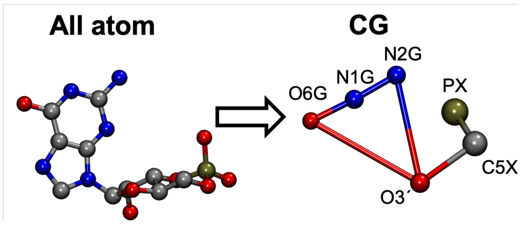

SIRAH name	q(e)	σ (Å)	ε (kcal/mol)
**PX**	-1.0	2.6000	0.2000
**C5X**	0.0	2.4080	0.1094
**O3'**	0.0	1.9080	0.1094
**O6G**	-0.4	1.6612	0.3100
**N1G**	0.2	1.8240	0.2600
**N2G**	0.2	1.8240	0.2600

Figure S1. Mapping of the cGMP form all atom to CG. According to SIRAH philosophy the equilibrium distance is according to experimental information measure from the PDB, no dihedral angles were added for the flexibility. Bonds are assigned a force constant of 41840 kJ/mol nm^2. Table with bead's names, partial charges and van der Waals parameters.

KHTEYMEFLKSVPTFQSLPEEILSKLADVLEETHYENGEYIIRQGARGDTFFIISKGTVNVTREMKRK
GKSPVATMVSKGEELFTGVVPILVELDGDVNGHKFSVSGEGEGDATYGKLTLKFICTTGKLPVPWP
TLVTTLGGGVQCFSRYPDHMKQHDFFKSAMPEGYVQERTISFKDDGNYKTRAEVKFEGDTLVNRI
ELKGIDFKEDGNILGHKLEYNYNSHNVYITADKQKNGIKANFKIRHNIEDGSVQLADHYQQNTPIG
DGPVLLPDNHYLSTQSALSKDPNEKRDHMVLLQFVTAAGITHGMDELYKSGLRSRADPVFLRTLG
KGDWFGEKALQGEDVRTANVIAAEAVTCLVIDRDSFKHLIGGLDDVSNKAYSKGEELFTGVVPILV
ELDGDVNGHKFSVSGEGEGDATYGKLTLKFICTTGKLPVPWPTLVTTLGGGVQCFSRYPDHMKQ
HDFFKSAMPEGYVQERTISFKDDGNYKTRAEVKFEGDTLVNRIELKGIDFKEDGNILGHKLEYNYNS
HNVYITADKQKNGIKANFKIRHNIEDGSVQLADHYQQNTPIGDGPVLLPDNHYLSTQSALSKDPNE
KRDHMVLLQFVTAAGITL

Figure S2. The primary sequence of CUTie2. Each domain of the sensor is shown in different color: CNBD (violet). linkers (grey). YFP (yellow) and CFP (cyan).

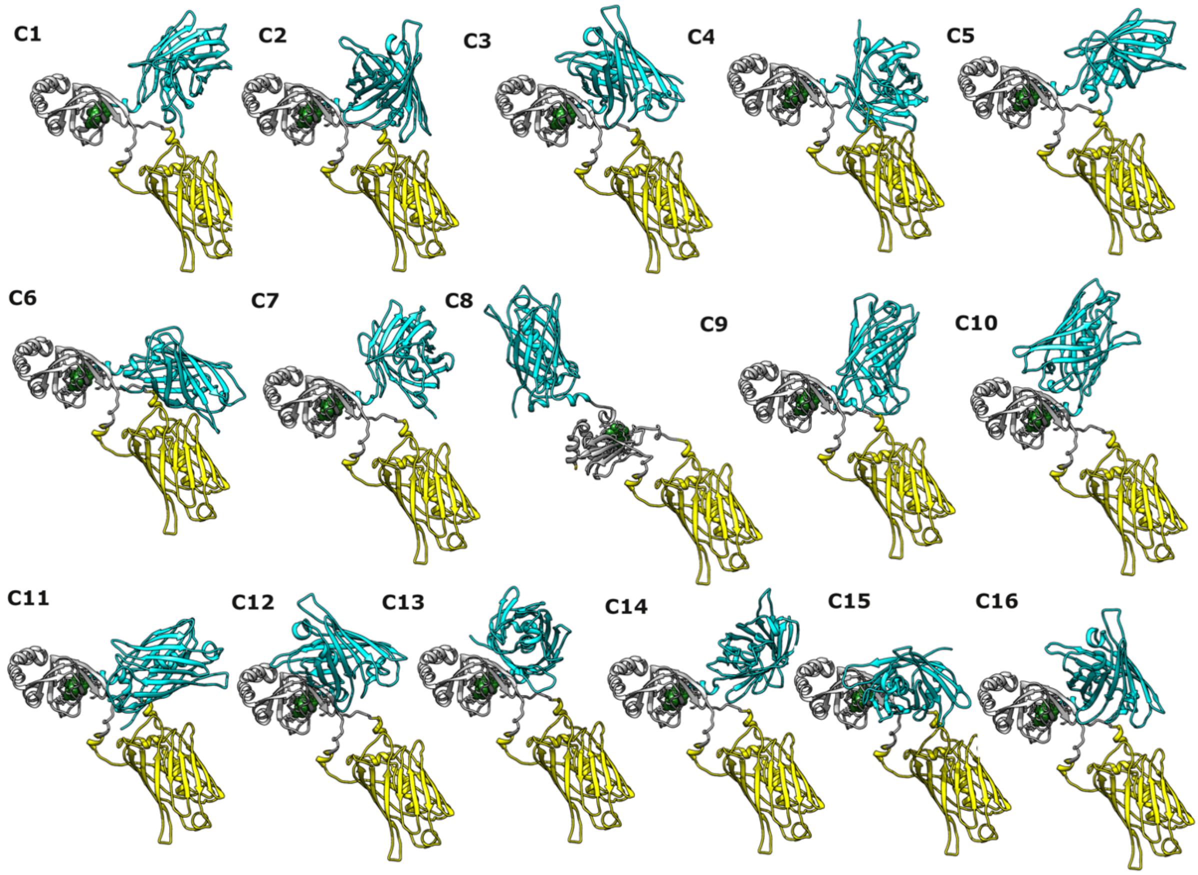
C1
C2
C3
C4
C5
C6
C7
C8
C9
C10
C11
C12
C13
C14
C15
C16

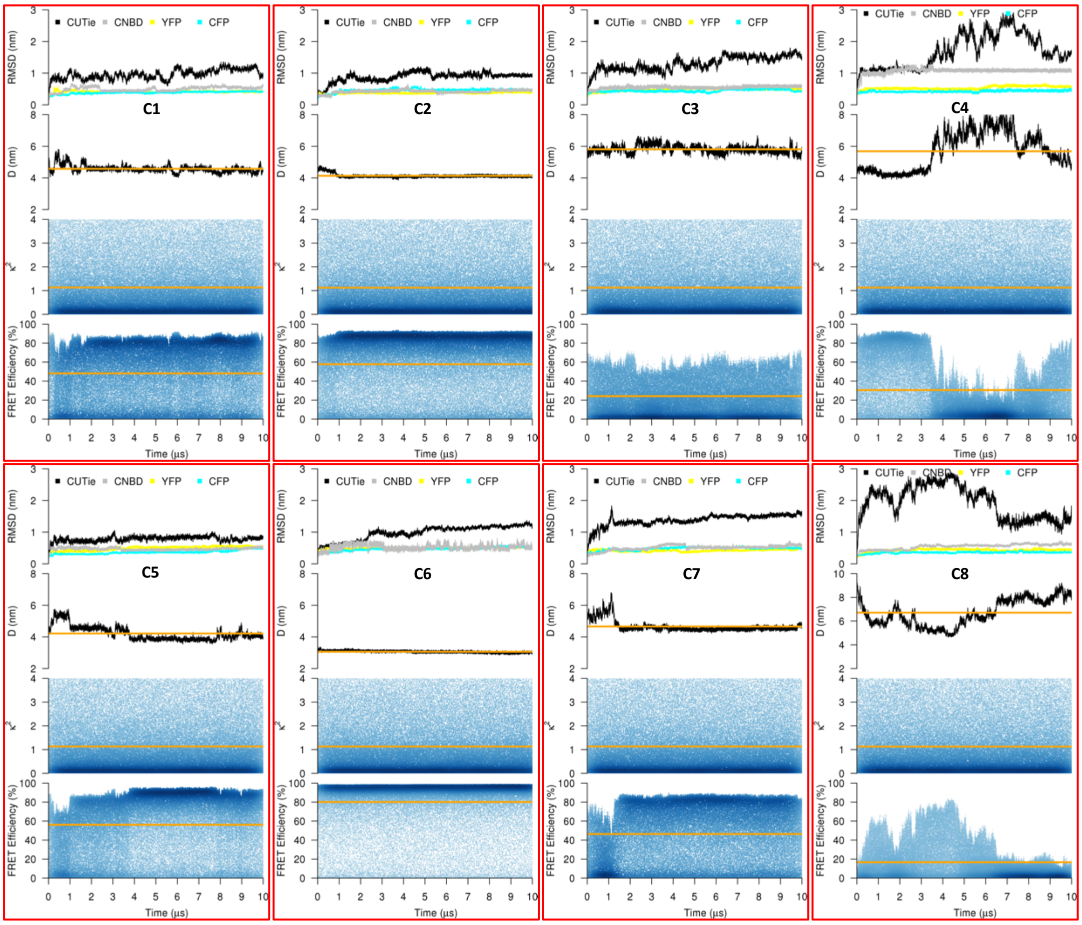
CUTie
CNBD
YFP
CFP
RMSD (nm)
D (nm)
κ²
FRET Efficiency (%)
Time (µs)
C1
C2
C3
C4
C5
C6
C7
C8

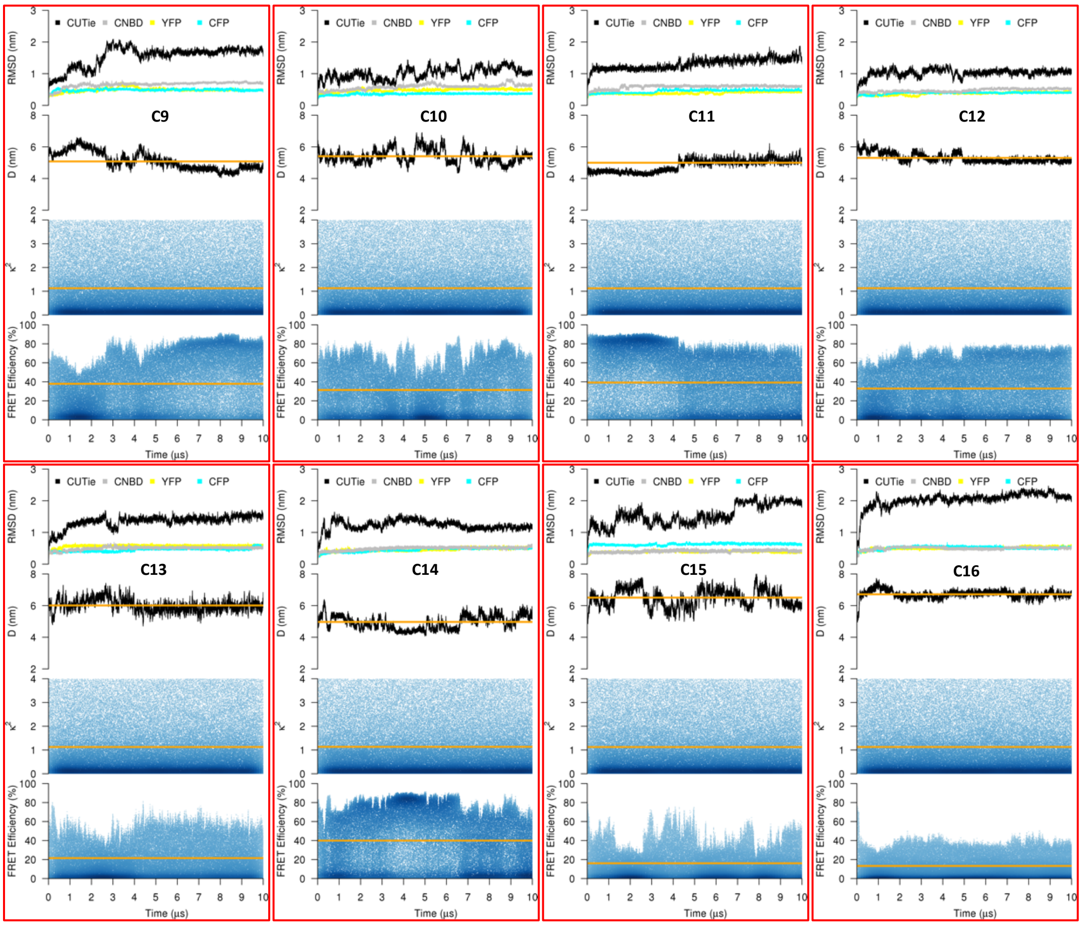

Figure S3. CG simulation of the 16 different conformers of the CUTie2 sensor making a cumulative time of 0.16 ms.

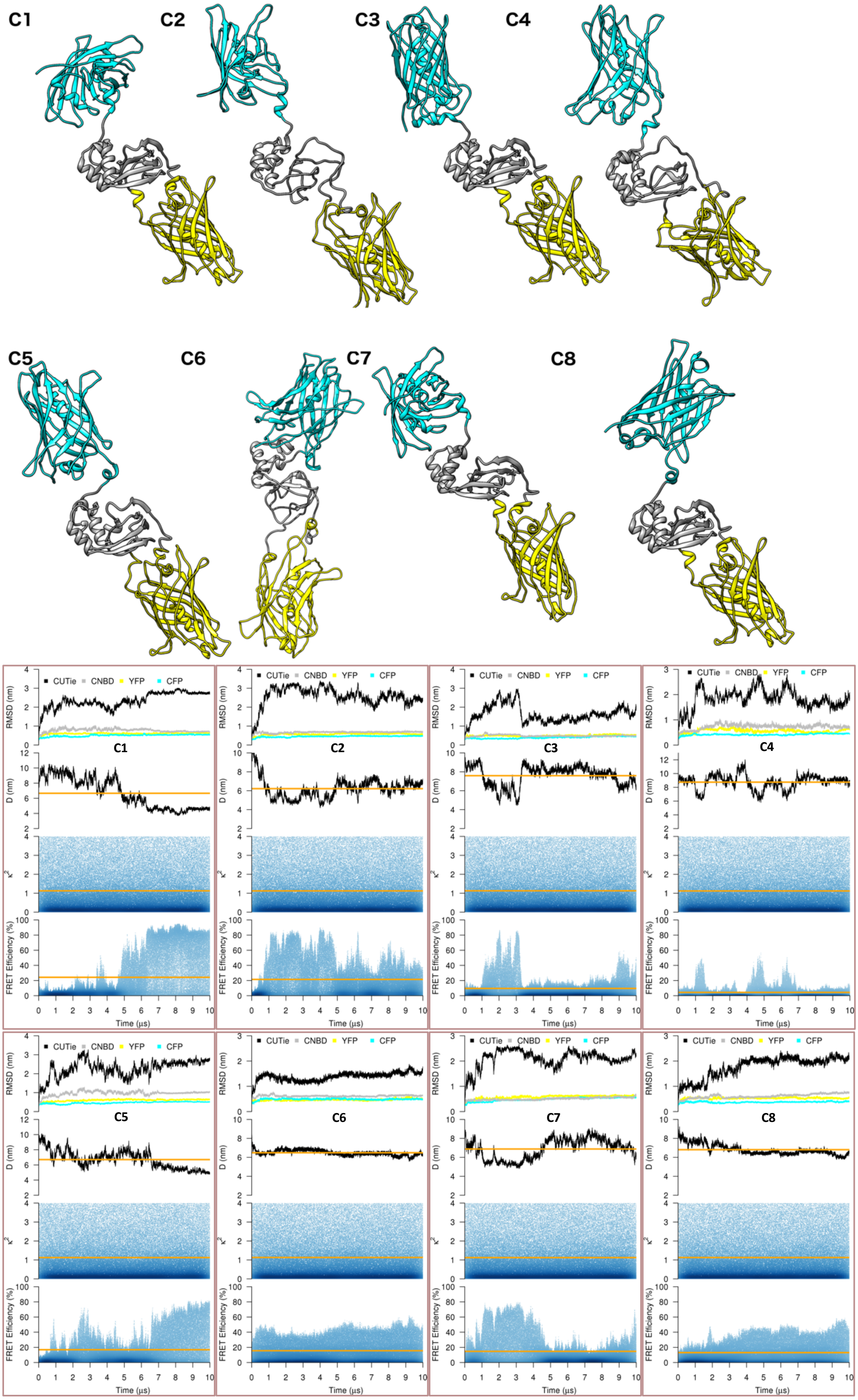

Figure S4. CG simulation of the 8 different conformers of the unbound CUTie2 sensor making a cumulative time of 80 µs.

Assay 3 (n=2)

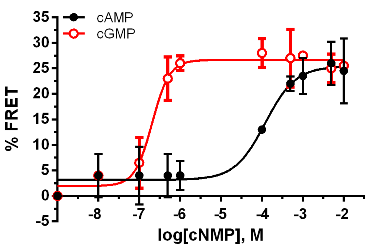

	cGMP	cAMP
**EC_{50}**	208 ± 39 nM	121 ± 11 μM
**Hill slope**	2.0	1.2
**R^2**	0.98	0.99

Assay 2 (n=2)

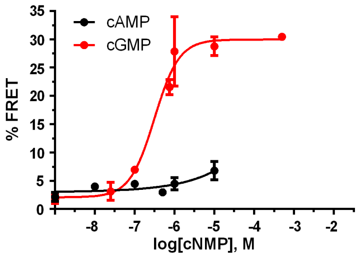

	cGMP	cAMP
**EC_{50}**	311 ± 83 nM	>> 10 μM
**Hill slope**	1.2	ND
**R^2**	0.98	0.67

Assay 1(n=1)

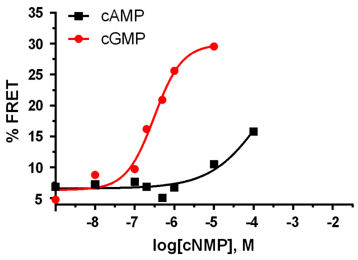

	cGMP	cAMP
**EC_{50}**	313 ± 90 nM	206 ± 97 μM
**Hill slope**	1.4	0.6
**R^2**	0.99	0.92

Average values from three independent experiments:

EC_{50} cGMP 277 ± 60 nM (Hill slope = 1.5 ± 0.4)
EC_{50} cAMP 167 ± 54 μM (Hill slope = 1.2, from Assay 3)
Selectivity (IC_{50} cAMP/cGMP) = 600-folds

Figure S5. Titration experiments of CUTie2 biosensor with cGMP and cAMP. Dose-response plots obtained for three independent assays with replicate samples indicated in parenthesis. The tables below the plots show the values for the different parameters evaluated. ND, not determined. The average value and standard deviation for the IC_{50}, Hill slope (if applicable) and the selectivity index from the three assays is shown at the bottom in blue fonts. Assay 3 is also shown in Fig. 3C as representative experiment.
