# Supplementary material for: CUTie2: The Attack of the Cyclic Nucleotide Sensor Clones
Source: Front Mol Biosci. 2021 Mar 11;8:629773. doi: 10.3389/fmolb.2021.629773 (PMC7991088; doi:10.3389/fmolb.2021.629773)
Supplement: Supplementary file 4 [file table2.docx]

CUTie2: The Attack of the cyclic nucleotide sensor clones

Florencia Klein^1,2^, Florencia Sardi^3^, Matías R. Machado^1^, Claudia Ortega^4^, Marcelo Comini^3^, Sergio Pantano^1,5*^

^1^ BioMolecular Simulation Group, Institut Pasteur de Montevideo, Montevideo, Uruguay. Mataojo 2020, CP 11400

^2^ Graduate Program in Chemistry, Facultad de Química, Universidad de la República, Uruguay.

^3^ Laboratory Redox Biology of Trypanosomes, Institut Pasteur de Montevideo, Montevideo, Uruguay. Mataojo 2020, CP 11400

^4^ Recombinant Protein Unit, Institut Pasteur de Montevideo, Montevideo, Uruguay. Mataojo 2020, CP 11400

^4^ Shanghai Institute for Advanced Immunochemical Studies, ShanghaiTech University, Shanghai 201210, China.

***Correspondence:**Sergio Pantano
spantano@pasteur.edu.uy

Keywords: CUTie2, Biosensor, FRET, Molecular Dynamics, Coarse-Grained, SIRAH.

Abstract

The detection of small molecules in living cells using genetically encoded FRET sensors has revolutionized our understanding of signaling pathways at the sub-cellular level. However, engineering fluorescent proteins and specific binding domains to create new sensors remains challenging because of the difficulties associated with the large size of the polypeptides involved, and their intrinsically huge conformational variability. Indeed, FRET sensors' design still relies on vague structural notions, and trial & error combinations of linkers and protein modules.

We recently designed a FRET sensor for the second messenger cAMP named CUTie (**C**yclic nucleotide **U**niversal **T**ag for **i**maging **e**xperiments), which granted sub-micrometer resolution in living cells. Here we apply a combination of sequence/structure analysis to produce a new-generation FRET sensor for the second messenger cGMP based on Protein Kinase G I (PKGI), which we named CUTie2. Coarse-grained molecular dynamics simulations achieved an exhaustive sampling of the relevant spatio-temporal coordinates providing a quantitative prediction of the FRET efficiency, as confirmed by *in vitro* experiments. Moreover, biochemical characterization showed that the cGMP binding module maintains virtually the same affinity and selectivity for its ligand that the full-length protein.

The computational approach proposed here is easily generalizable to other allosteric protein modules, providing a cost effective-strategy for the custom design of FRET sensor.

# Introduction

The development of genetically encoded sensors based on Föster resonance energy transfer (FRET) effect is a well-established methodology for the non-invasive and real-time study of a plethora of cellular events (Nikolaev and Lohse, 2006; Berrera et al., 2008; Meng and Sachs, 2011; Calamera et al., 2019).

In general, FRET sensors are composed of a molecular detector, which undergoes a conformational change upon a given signal. A couple of fluorophores suitable for FRET and linked to convenient domains of the detector module complete the sensor architecture. Two fluorophores are a suited FRET pair if there is a substantial overlap between the spectra of emission and absorption of the donor and acceptor, respectively (Lindenburg and Merkx, 2014). The use of spectral variants of the Green Fluorescent Protein (GFP) as FRET pairs opened the possibility to generate genetically encoded sensors for monitoring intracellular signaling (Zaccolo et al., 2000). In particular, FRET sensors applied to the study of cyclic nucleotides (CNs) have been instrumental for dissecting molecular details of the corresponding signaling pathways, and several protein architectures have been reported to work as FRET sensors for CNs (Sprenger and Nikolaev, 2013; Pendin et al., 2016).

In the case of CNs, most often the detector is a cyclic nucleotide-binding domain (CNBD) genetically fused to suitable spectral variants of GFP. When the intracellular concentration of cyclic nucleotides rises, they bind to the CNBD, triggering an allosteric conformational change that modifies both fluorophores' relative distance and/or orientation, translating into a change of the FRET signal (Lindenburg and Merkx, 2014). Despite the proven usefulness of genetically encoded sensors, very little work has been done to advance on methods to systematically engineer FRET sensor achieving quantitative predictions of FRET efficiency (Stangherlin et al., 2014). Indeed, the design of novel sensors is guided by a limited structural knowledge of the CNBDs. Such a procedure often results in an undetermined and frustrating number of trial & error attempts.

We were among the firsts to apply modeling techniques to improve cAMP FRET sensors (Lissandron et al., 2005) and provide simple structure-based rules to guide the design of CN sensors (Machado and Pantano, 2015). More recently, we showed that the combination of modeling techniques with coarse-grained (CG) simulations effectively led to a quantitative prediction of the FRET change upon raising cAMP concentrations. Moreover, the rational design of the so-called CUTie sensor produced for the first time a FRET sensor targetable to different subcellular compartments that unveiled the presence of unexpectedly small cellular (nano)domains regulated by cAMP (Chao et al., 2019). Quantitative predictions of the FRET efficiency of the CUTie sensor were achieved thanks to the comprehensive sampling provided by CG simulations. As confirmed experimentally, our cost-effective approach achieved an exhaustive conformational sampling of the space accessible to the fluorescent proteins linked to the cAMP-binding domain of the regulatory subunit of the RIIβ isoform of Protein Kinase A (Surdo et al., 2017). Here, we aimed to prove the general validity of our approach by designing and testing a FRET sensor for cyclic guanosine 3'-5'-monophosphate (cGMP). This latest generation sensor will be referred as CUTie2 hereafter.

The second messenger cGMP is involved in several downstream intracellular signaling pathways. Among others, cGMP plays a central role in regulating the cardiomyocyte function (Perera et al., 2015; Bork et al., 2019; Zhao et al., 2020) and is involved in neurological diseases (Knott et al., 2017; Schepers et al., 2019). In mammalian cells, the production of cGMP is regulated by the binding of Nitric oxide (NO) to soluble guanylyl cyclases and natriuretic peptides, that activate particulate guanylyl cyclases GC-A and GC-B; while, while its degradation is tightly controlled by eight out of the eleven isoforms of Phosphodiesterases (Friebe et al., 2020).

The development of cGMP sensors pose the challenge of achieving simultaneously high affinity for cGMP vs. cAMP, which is normally present at higher concentrations in overlapping subcellular compartments (Stangherlin and Zaccolo, 2012). Nevertheless, a number of cGMP sensors have been reported (Sprenger and Nikolaev, 2013). The most frequently used sensors are based on mammalian proteins, which, due to their relatively high EC_50_ (Cygnet2.1 and cGI-500 EC_50_ = 0.5 μM, cGES-DE5 EC_50_ = 1.9 μM) (Honda et al., 2001; Nikolaev et al., 2006; Russwurm et al., 2007) they are useful in environments with high concentrations of cGMP. So far, the sensor with higher affinity for cGMP has reported EC_50_ ~ 40 nM (Niino et al., 2009; Calamera et al., 2019).

Therefore, it is highly desirable to count with a targetable sensor with similar characteristics to those available for cAMP.

The design of allosteric sensors requires the knowledge of the structure and conformational landscape accessible to the protein. Schematically, the binding site of CNBDs is constituted by an 8-stranded β-barrel, which is flanked by two helices commonly designated as helix A and B (Figure 1A). In most CNBDs, a C-terminal helix (helix C) acts as a lid that closes the binding pocket in the presence of CNs (Berman et al., 2005). In absence of the ligand, the C helix is highly mobile to the point it can’t be solved by X-ray crystallography. This conformational behavior makes this family of protein structures good candidates for FRET sensors, and the C-terminal an obvious place to insert a fluorescent protein. During the design of the original CUTie sensor, we found that the loop connecting β-strands 4 and 5 (named 4-5 loop) is poorly conserved, with frequent insertions in different cAMP binding domains (see Figure 1A). This suggested that the 4-5 loop is structurally permissive to insert a fluorescent molecule without altering CNBD´s characteristics (Surdo et al., 2017). Therefore, we further explore the possibility of engineering another CNBD to generate a FRET sensor for cGMP. With this aim, we focused on the human Protein kinase G (PKG), which has two isoforms (I and II) (Butt et al., 1993; Hofmann et al., 2009), sharing the same overall architecture and CNBD organization (Vaandrager et al., 2005; Campbell et al., 2016).

In-vitro experiments showed that the CUTie2 sensor developed in-silico maintains a nearly native affinity (EC_50_ = 277 nM) and over two orders of magnitude selectivity for cGMP vs. cAMP, reaching a maximal FRET change of 26%. Since CUTie2 maintains the same architecture of its cognate CUTie, it might offer the possibility to dissecting simultaneously the submicrometric coexistence of cAMP and cGMP signals. Moreover, because of the global similarity of CNBDs, the procedure illustrated here should be generalizable to arbitrary proteins of this class.


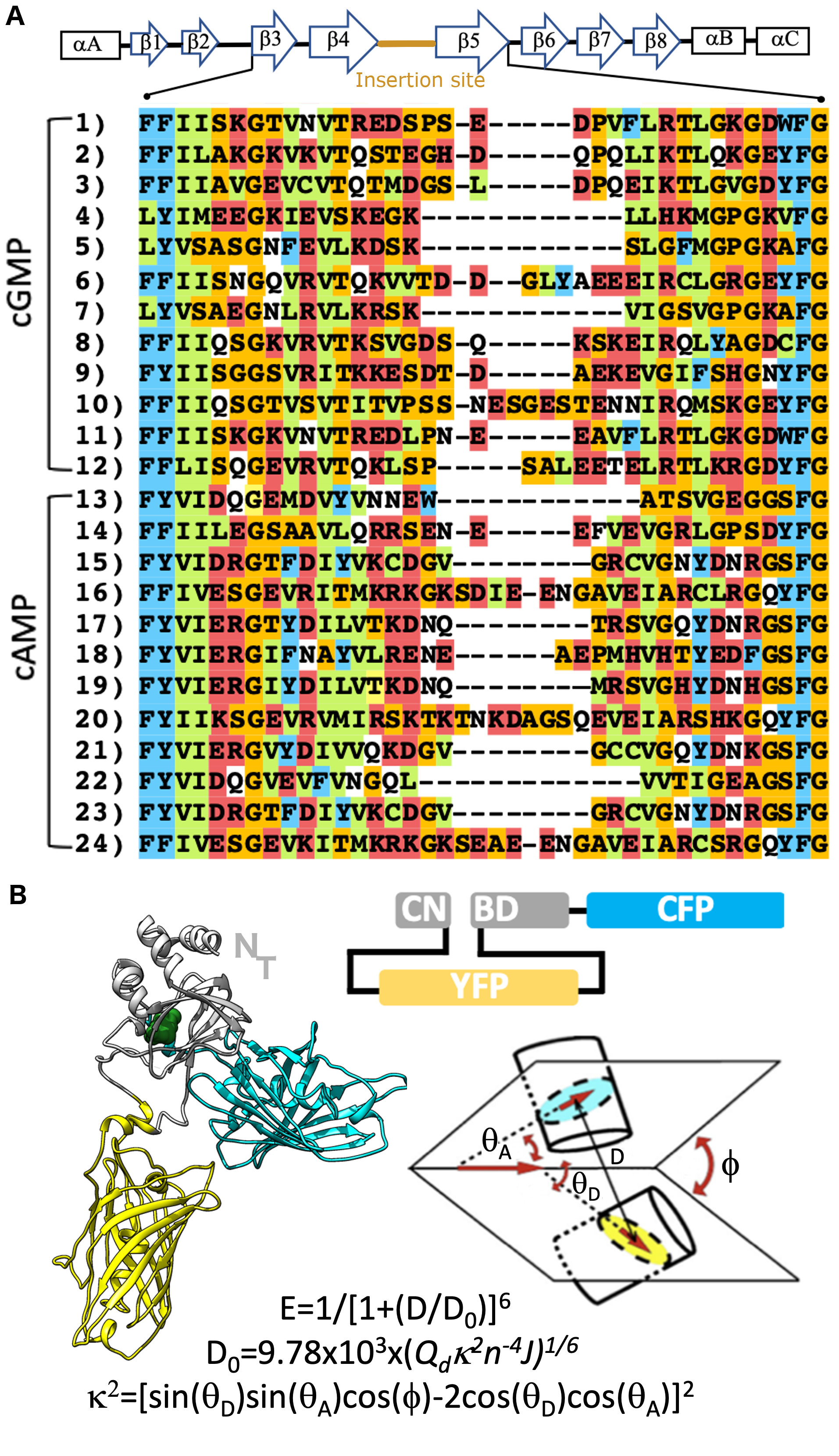


**Figure 1.** Design of CUTie2. **A)** Global secondary structure of a CNBD and multiple sequence alignment of related domains. Rectangles and arrows are used to indicate α-helices and β-strands. Sequences 1 to 12, and 13 to 24 correspond to cGMP and cAMP binding proteins, respectively. These correspond to the UNIPROT codes, for the cGMP binding domains: 1) Q13976, 2) Q13237, 3) A0A444U9Q8, 4) A0A443SJC3, 5) B0X970, 6) A0A4Y2N1F1, 7) A0A4Y2BIM3, 8) A0A419PY19, 9) A0A2P8Y1R0, 10) A0A3R7JPE0, 11) A0A444UAH2, 12) A0A0M4ENX8 and the cAMP binding modules: 13 & 14) P00514, 15 & 16) P12369, 17) A0A2J8TG41, 18) E0VRT6, 19 & 20) A0A091CJC2, 21) A0A556UFG6, 22) A9QQ52, 23 & 24) A0A384A0R8. Aminoacids are colored by physicochemical character according to CLUSTAL.

**B)** Cartoon representation and domain organization of the CUTie2 sensor. Notice that the N-terminal of the protein remains free to be eventually fused to arbitrary targeting domains. The schematic architecture of the sensor and definition of the geometric factors and equations used to calculate the FRET signal are provided on the right.

# Methods

In silico design.

To find a suitable insertion point for the first fluorescent module we perform a multiple sequence alignment of protein containing CNBDs. To ensure the generality of our results, we consider sequences from mammals, insect, fish, arachnid, crustacean and annelid (Figure 1A). CNBD protein sequences are retrieved from the UniProt database (Bateman, 2019, https://www.uniprot.org) and aligned using ClustalW in the MPI Bioinformatics Toolkit (Bioinformatics Toolkit).

3D starting conformations are built on the base of X-ray structures of PKGIβ protein (PDBid: 4KU7, residues K223 to Y351 (Huang et al., 2014)). Because of practical reasons, both fluorescent modules (Yellow and Cyan Fluorescent Proteins) were modeled using the X-ray structure PDBid: 1QYO (Barondeau et al., 2003). This corresponds to a triple mutation of GFP, without the chromophore moiety. This variant presents the typical GFP folding and obviates the need to parameterize CG chromophores. This shortcut is well tolerated within the coarseness of our approach. Initially, the CNBD, linkers and the fluorescent module corresponding to the YFP were manually positioned at distances compatible with covalent bonds and ligated with Chimera (Pettersen et al., 2004). Subsequently, the CFP module is attached to Y351 in the CNBD. Once a model is complete, its structure is energy optimized by CG simulations (see below). Different initial conformers are generated by arbitrarily rotating torsional angles between the CNBD and the CFP module. For simulations in the cGMP-free protein, a new set of conformers are generated in which the ligand is removed before starting the MD simulations.

CG simulations.

CG molecular dynamics (MD) simulations are performed using the SIRAH 2.0 force field under the conditions reported in (Machado et al., 2019). The interaction parameters for CG cGMP and the simulation protocol are reported in supplementary material (Figure S1). Briefly, atomistic models are mapped to CG using SIRAH Tools (Machado and Pantano, 2016) and solvated with CG water up to 2 nm beyond the last bead in each direction. Explicit CG ions are added to set an ionic strength of 15 mM. Although simulation boxes are different for each protomer, systems typically contained 50000 beads, roughly representing half a million atoms. The simulation protocol consists of 5000 steps of unrestrained energy minimization, and five MD equilibration steps in NPT ensemble at 1 bar using the v-rescale thermostat and Parrinello-Rahman barostat:

1. Solvent equilibration by 5 ns at 10 K and time step of 2.0 fs
2. 40 ps at 300 K and time step of 2.0 fs.
3. 400 ps at 300 K with a time step 20 fs.
4. 400 ps at 300K with a time step 20 fs and positional restrains of 1000 kJ mol^-1^nm^-2^ on the whole protein.
5. Same as the previous one but for 5 ns.
6. Production simulations for 10 μs.

Each equilibration step starts from 0 K temperature (i.e, velocities are zeroed at each restart). We generated 16 different initial conformers in cGMP-bound and 8 in cGMP-free conformation, totalizing a cumulative simulation time of 0.24 ms. Snapshots are recorded every 100 ps for analysis.

Non-bonded interactions are calculated with a 1.2 nm cutoff and PME method (Darden et al., 1993; Essmann et al., 1995) for long-range electrostatics. Simulations are performed using GROMACS 4.6.7 (http://www.gromacs.org).

Analysis of the trajectories.

The FRET efficiency (E) depends only on the inter-fluorophore distance (D) and the relative orientation of the chromophores (commonly named orientational factor, or simply κ^2^). The change in FRET is calculated by subtracting the average FRET values in cGMP-bound and cGMP-free conformations. D is estimated as the distance between the geometric centers of CFP and YFP, while κ^2^ factor is calculated from the dipoles defined between the geometric center of the fluorescent protein and the Cα of Ser147 in each fluorescent module (Figure 1B). This agrees with the orientation of the dipole moment estimated from DFT calculations for a variety of chromophores in different fluorescent proteins (Rosell and Boxer, 2003). Root Mean Square Deviations (RMSD) are calculated on the Cα beads.

The FRET efficiencies in cGMP-bound and -free states are calculated as the running average with sliding window of 100 ns over the concatenated trajectories. Obviously, the final averages are independent of the order in which the trajectories are concatenated. The standard deviations in the simulated FRET are calculated after convergence (i.e., after a cumulative time = 0.04 ms).

Experimental.

All chemicals and reagents used are of analytical grade and purchased from SIGMA. The DNA plasmid is synthetized by Genscript and sequenced by the Molecular Biology Unit, Institut Pasteur of Montevideo.

The recombinant form of CUTie2 is expressed as an N-terminally 6 His-tagged protein using the vector pET28a(+) and *Escherichia coli* Rosetta (DE3) as heterologous expression host. The transformed bacteria are grown at 37°C in 2YT medium containing 50 µg/mL Kanamycin and 34 µg/mL Chloramphenicol until an optical density of 0.8. Thereafter, 0.5 mM IPTG (Euromedex) is added to the culture and incubation extended for 16 hours at 20°C. After centrifugation at 6000 *g* for 30 min at 4°C, the cell pellet is resuspended in 50mM Tris-HCl pH 8.0, 300 mM NaCl, 20mM Imidazole (Buffer A), containing a EDTA-free protease inhibitors cocktail (ROCHE) and Lysozyme (end concentration 1 mg/mL). The cell suspension is subjected to sonication and the debris removed by centrifugation at 16,000 *g* for 1 hour at 4°C. The clarified extract is applied to a 1 ml HisTrap column (GE Healthcare) and upon washing in Buffer A, the His-tagged protein is eluted with buffer A containing 500 mM Imidazole. The fractions containing the cGMP sensor protein are pooled and concentrated by centrifugation (5000 *g* at 4°C) in a Vivaspin-20 filter (30 kDa cutoff), and further polished by a Superdex G-200 size exclusion chromatography (10/30 column) run in 50mM Tris-HCl (pH 8.0), 500 mM NaCl.

Protein concentration is measured at 280 nm, where ε_280_ for CUTie2 60.990 M^-1^ cm^-1^. In a Hellma® fluorescence cuvette (total reaction volume of 120 µl), the sensor (100 nM) is treated with different concentrations of cGMP or cAMP (0 nM – 10 mM) in PBS buffer containing 1 mM EDTA pH 7.0. Upon addition of cGMP or cAMP, the fluorescence emission spectrum (λexc = 435 nm, λem = 460 - 600 nm) of CUTie2 is recorded in a Cary Eclipse fluorimeter every 1 min during 10 min and using slit widths of 5 nm and a PMT of 780. The fluorescence intensity (FI) values corresponding to CFP (485 nm) and YFP (527 nm) in the absence (C0) or presence of different concentrations of the corresponding cyclic nucleotides (Cx) are used to calculate the percentage FRET as follows:

$$\text{\%}FRET=\left[ \left( \frac{{FI}_{Cx}^{527nm}}{{FI}_{Cx}^{485nm}} \right)-\left( \frac{{FI}_{C0}^{527nm}}{{FI}_{C0}^{485nm}} \right) \right]\times100\text{\%}$$

The % FRET *vs.* Log[cNMP] is plotted, fitted to non-linear regression equations and statistically analyzed using the GraphPad Prism 6 Software (San Diego, CA, USA). Three independent titration experiments are performed with three different batches of the recombinant sensor.

# Results and discussion.

- 1. In silico design

In order to define the structure of the cGMP sensor, we aligned the protein sequences reported in Figure 1A. We identify the 4-5 loop as a suitable insertion site in cGMP binding domains. The second fluorescent module is added to the C-terminal of the protein, after the end of the C-helix. The scheme of the construct and a molecular representation of CUTie2 are shown in Figure 1B.

The module corresponding to YFP is inserted within the cGMP-CNBD. It substitutes the original amino acids Asp_286_-Ser-Pro-Ser-Glu_282_ in the 4-5 loop by the linkers used in the CUTie sensor, as previous MD simulations suggested they avoid spurious distortions in the binding domain (Surdo et al., 2017). The substitution of the original amino acids in the sequence is not expected to alter significantly the conformation of the CNBD as they are not determined in the X-ray structure. This assumption could be verified *a posteriori* (see below). The CFP is linked directly to the C-terminal of the CNBD. As a proof of concept of our methodology, we choose the domain β of human PKG I, for which the 3D structure and cGMP binding characteristics are well defined (Huang et al., 2014). A schematic representation of the CUTie2 sensor and its primary sequence are shown in Figure 1B, and supplementary Figure S2, respectively.

Having the primary sequence and structural knowledge of the protein modules, it is possible to construct 3D representations of the sensor. However, a quantitative prediction of the FRET efficiency requires a complete sampling of all relevant collective variables. In this regard, it may be important to recall that the FRET effect is a dipole-dipole interaction, which rapidly decays as the 1/D^6^ (Figure 1B), where D is the distance between chromophores. In practical terms, 10 nm is considered as an upper limit for the distance of interaction between two fluorescent proteins. On the other hand, the numerical value of κ^2^ ranges from 0 to 4 (see definition in Figure 1B). Therefore, a proper sampling of the relevant coordinates to quantify the FRET effect must achieve a complete sampling of D and κ^2^ within the above range. In principle, MD simulations can be used to this aim. However, the computational cost of such simulations is prohibitive. Hence, we sorted out to use the accurate and topologically unbiased SIRAH force field for CG simulations (Machado et al., 2019). Different starting conformers are prepared in the cGMP-bound and -free states and simulated for 10 μs each.

Figure 2 shows the main determinants of a simulation of cGMP-bound to CUTie2. The relative mobility of each protein module (CNBD and the two fluorescent variants), and the whole construct is characterized in terms of their RMSD from their initial positions. Individual domains show quite stable and relatively low RMSD values. However, the CUTie2 sensor as a whole shows excursions up to 2 nm with stability regions in time-windows of about 2 μs (Figure 2A). Those flat RMSD regions show a rough correspondence to the distance between chromophores (Figure 2B), indicating that the stable regions in the global RMSD can be identified with large-scale movements of the fluorescent modules. Indeed, the distance between chromophores vary from 5 nm up to 8 nm in this particular staring conformer. Calculation of κ^2^ shows that the simulation samples the complete spectrum of values with a much faster dynamics (Figure 2C). The reason for this difference is rather obvious, while significant variations in D imply the motion of entire protein modules, the dynamics of κ^2^ is related to rotations of torsional dihedrals associated to the protein backbone, which happen spontaneously in the nanosecond timescale at room temperature. The combination of both collective variables as detailed in Figure 1B allows for a theoretical estimation of the FRET illustrated in Figure 2D. Because of marked difference in the dynamics of D and κ^2^, the FRET efficiency is mostly correlated to the inverse of D.


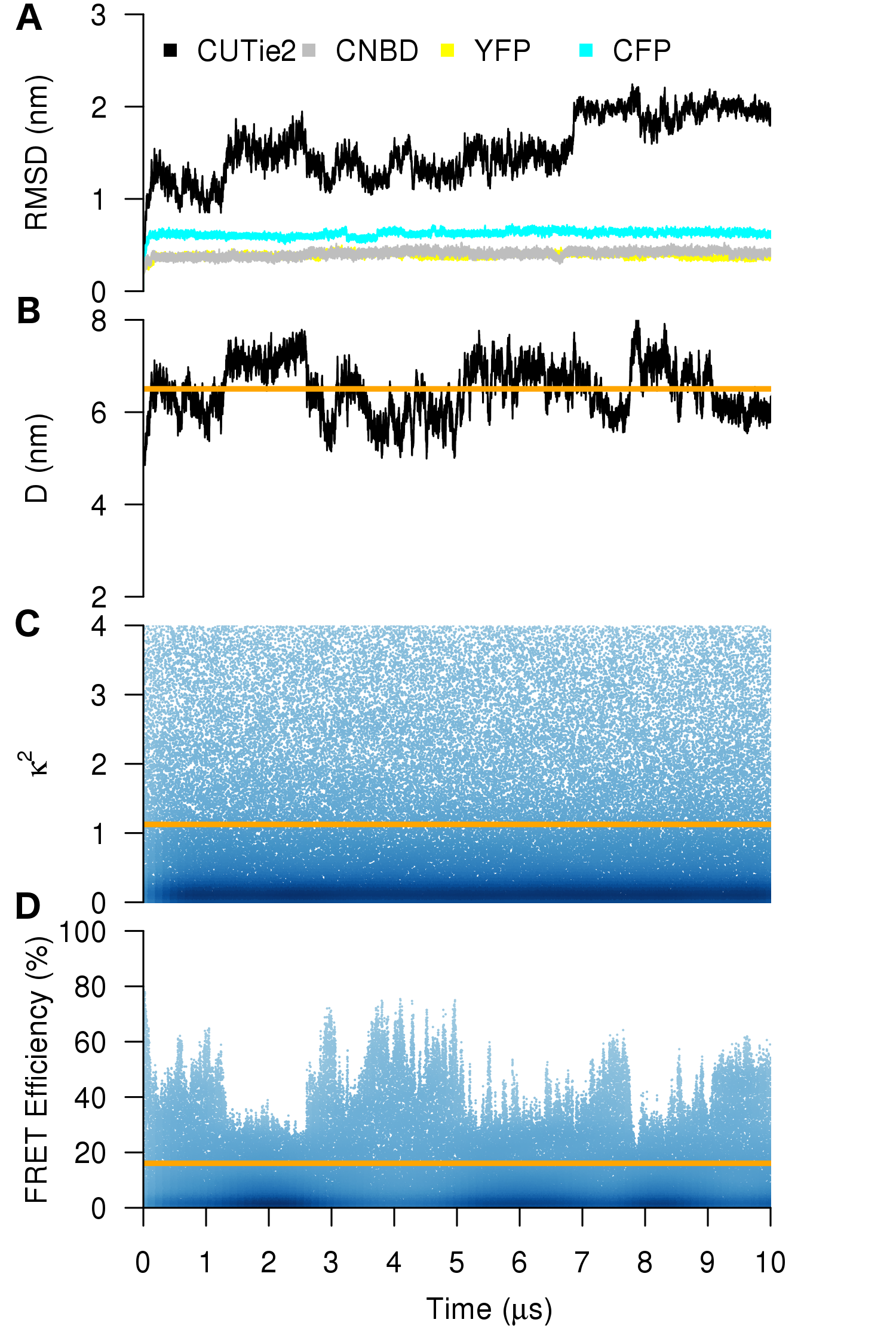


**Figure 2.** CG simulation of the CUTie2 sensor. **A)** Instantaneous RMSD of one randomly chosen conformer in cGMP-bound conformation . Black, gray, yellow, and cyan correspond to the whole molecule, the CNBD, the YFP, and the CFP modules, respectively. **B to D)** Distance between chromophores, κ^2^, and FRET efficiency. The orange line in each panel corresponds to the average over the entire trajectory.

Experimentally, the FRET efficiency averages on a wide number of conformations and within time windows of minutes. Intuitively, a proper comparison between simulated and experimental FRET efficiencies requires performing simulations of the sensor in cGMP-bound and –free states, calculating the average FRET efficiencies in both states and, then subtract both values to obtain the variation in FRET efficiency upon cGMP binding. For such comparison to be meaningful, one should assume a broad sampling of the relevant variables, namely, D and κ^2^. While Figure 2B suggests that this is true for κ^2^, D is still limited to a restricted range (Figure 2C).

Therefore, we generate two sets of simulations with different starting conformers in cGMP-bound and –free states. After each 10 μs simulation, we concatenate the trajectories and calculate the running average of the FRET efficiency (Figure 3A). New conformers are generated until convergence is reached. In the cGMP-bound case we generate a total of 16 different conformers for a cumulative time of 0.16 ms (see supplementary Figure S3). For cGMP-free case, only 8 conformers are dimmed necessary since in the absence of cGMP the two fluorophores sample preferentially higher D distances (see supplementary Figure S4).

The average change in FRET efficiency resulted in 22% (s.d. 2, Figure 3A).

To further illustrate the conformational sampling we plot the FRET efficiencies of the different starting conformers as a function of D and κ^2^ provides a breakdown of sampling achieved (Figure 3B, only the cGMP-bound case is shown for brevity). The whole set of points covers the range D<10 nm and 0> κ^2^ >4. The spreading of the distribution clearly shows that some conformers remain trapped in relatively narrow conformational regions, while others can widely explore the D variable. Using the backmapping capability of the SIRAH force field (Machado and Pantano, 2016), we produce pseudo atomistic representative snapshots from three different starting conformations. The violet molecule (indicated with the letter “a” in Figure 3B) remains blocked in a high FRET configuration, while the red molecule (indicated with “b” in Figure 3B) explores a much wider range. In contrast, the cyan molecule (indicated with “c” in Figure 3B) spends most of the time with the two fluorescent modules far apart from each other, making little contributions to the global FRET. It is also interesting to notice that, because of the topology of the sensor, there is a region of values of D between 3 to 4 nm, which is structurally forbidden.


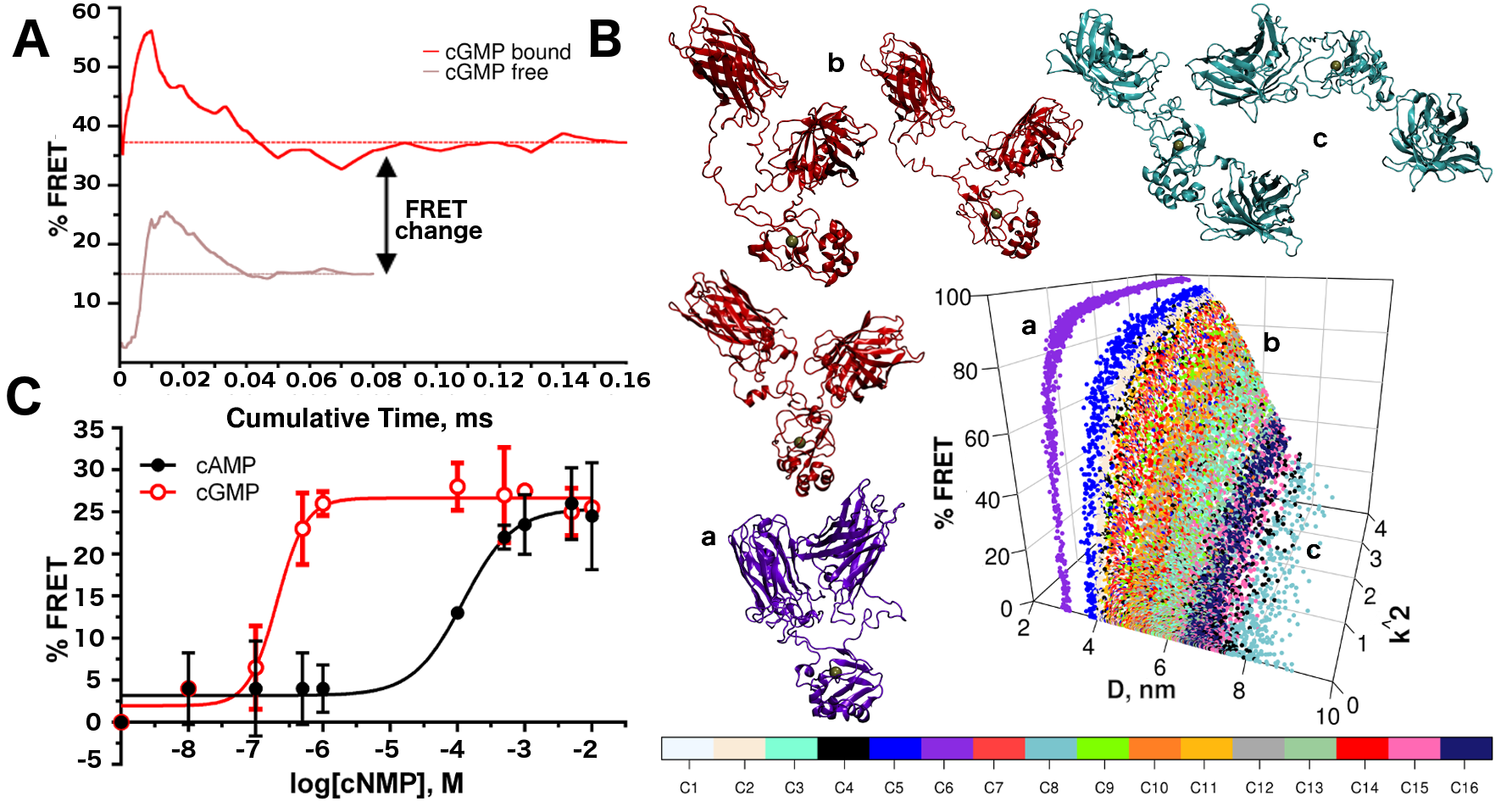


**Figure 3.** FRET efficiency and experimental validation. **A)** Averaged FRET values calculated from different conformers vs. simulated time for cGMP-bound and -free sets of simulations. **B)** Assessment of the completeness of the conformational sampling for the cGMP-bound simulations. The FRET efficiency is reported as a function of D and κ^2^ for each of the conformers (indicated by different colors). Six particular conformers taken from 3 trajectories are shown in cartoon representation to illustrate the conformational dispersion achieved during the simulations. Gray spheres indicate the cGMP binding sites. The letters in lower case indicate the position of the corresponding conformer in the graph. **C)** Representative concentration-response plot for recombinant CUTie2 titrated with different concentrations of cGMP and cAMP (0-10 mM). The data were fitted to the Boltzmann equation (R^2^ ≥ 0.98).

Experimental validation.

In order to provide experimental confirmation of the *in silico* predictions, the recombinant form of the CUTie2 sensor was expressed and the FRET signal measured *in vitro* for cGMP and cAMP in three independent experiments (see supplementary Figure S5).

As shown in Fig. 3C, the maximal FRET efficiency obtained at saturating concentrations of cGMP (FRET ≥26%) compares well with the computational predictions. Moreover, the effective concentration of cGMP that triggers a 50% FRET response of CUTie2 (EC_50_) to cGMP was 277 ± 60 nM, which nicely matches the value reported for this CNBD by using competition fluorescence polarization (Huang et al., 2014). Interestingly, the Hill´s slope estimated from the three independent cGMP titration assays with CUTie2 is 1.5 ± 0.4, which is within the range of values reported for PKG I (Huang et al., 2014) and a related FRET sensor (Honda et al., 2001), and may suggest a certain cooperative effect upon ligand binding. As mentioned before, the selectivity of the sensor for cGMP is essential for *in vivo* applications since in many subcellular compartments the cAMP concentration is higher than that of cGMP (Stangherlin and Zaccolo, 2012). In this regard, CUTie2 titration with cAMP revealed an EC_50_ of 121 ± 11 µM, which shows that the sensor has a >400-fold higher selectivity for cGMP.

# Discussion and Conclusions

This manuscript presents a computational methodology for the design and quantitative prediction of FRET efficiency of genetically encodable sensors for cGMP. The agreement with experimental data obtained here, and with the previously published CUTie sensor for cAMP (Surdo et al., 2017) supports the general validity and transferability of our approach. Within the standard deviations of FRET changes obtained by the experimental and computational determinations, our approach could be considered quasiquantitatively accurate. The capacity of both fluorescent modules to produce FRET at cGMP concentrations comparable to those measured for the intact protein strongly suggests that both the fluorescent proteins and the CNBD adopt their expected folding. This fact is not entirely obvious if we consider that YFP is inserted within the folding of the CNBD. In this regard, it may be worth mentioning that peptide linkers are present between the CNBD and YFP sequence (supplementary Figure S2). These linkers are the same used in the CUTie sensor. The marked differences in sequence conservation in the region of the 4-5 loop shown in Figure 1A and the correct functioning of CUTie and CUTie2 strongly suggest that these linkers are well suited for inserting a fluorescent module within the 4-5 loop of any CNBD. The equivalence in cGMP affinity and cAMP selectivity in relation to the intact CNBD further highlight this fact. This opens the possibility to generate a portfolio of sensors with different ligand affinities based on other biochemically well-characterized CNBDs or other ligand-specific domains that undergo a similar allosteric-dependent conformational change. CUTie and CUTie2 share the common feature of having a free N-terminal that can be fused to arbitrary targeting proteins/sequences. Namely, both can be regarded as FRET tags that can be targeted to different subcellular localizations determined by the accompanying protein or targeting signal. In summary, this new sensor holds great potential to unravel simultaneous signaling pathways with submicrometric resolution and high cyclic nucleotide selectivity in living cells.

9 ACKNOWLEDGMENTS

F.K. and F.S. acknowledge the support of Agencia Nacional de Investigación e Innovación (ANII), Uruguay (PhD fellowships POS_NAC_2018_1_151183 and POS_FMV_2015_1_1005325, respectively). F.K. acknowledge the Graduate Program in Chemistry (PEDECIBA), Facultad de Química, Universidad de la República, Uruguay. S.P. M.R.M. & M.A.C. belong to the Uruguayan National System of Researchers (SNI) from ANII. The Tesla K40 used for this research was donated by the NVIDIA Corporation. This work was partially supported by FOCEM (MERCOSUR Structural Convergence Fund), COF 03/11.

**References:**

Barondeau, D. P., Putnam, C. D., Kassmann, C. J., Tainer, J. A., and Getzoff, E. D. (2003). Mechanism and energetics of green fluorescent protein chromophore synthesis revealed by trapped intermediate structures. *Proc. Natl. Acad. Sci. U. S. A.* 100, 12111–12116. doi:10.1073/pnas.2133463100.

Bateman, A. (2019). UniProt: A worldwide hub of protein knowledge. *Nucleic Acids Res.* 47, D506–D515. doi:10.1093/nar/gky1049.

Berman, H. M., Ten Eyck, L. F., Goodsell, D. S., Haste, N. M., Kornev, A., and Taylor, S. S. (2005). The cAMP binding domain: An ancient signaling module. *Proc. Natl. Acad. Sci. U. S. A.* 102, 45–50. doi:10.1073/pnas.0408579102.

Berrera, M., Dodoni, G., Monterisi, S., Pertegato, V., Zamparo, I., and Zaccolo, M. (2008). “A Toolkit for Real-Time Detection of cAMP: Insights into Compartmentalized Signaling,” in *Handbook of Experimental Pharmacology* (Springer), 285–298. doi:10.1007/978-3-540-72843-6_12.

Bioinformatics Toolkit Available at: https://toolkit.tuebingen.mpg.de/ [Accessed August 23, 2020].

Bork, N. I., Molina, C. E., and Nikolaev, V. O. (2019). CGMP signalling in cardiomyocyte microdomains. *Biochem. Soc. Trans.* 47, 1327–1339. doi:10.1042/BST20190225.

Butt, E., Geiger, J., Jarchau, T., Lohmann, S. M., and Walter, U. (1993). The cGMP-dependent protein kinase-gene, protein, and function. *Neurochem. Res.* 18, 27–42. doi:10.1007/BF00966920.

Calamera, G., Li, D., Ulsund, A. H., Kim, J. J., Neely, O. C., Moltzau, L. R., et al. (2019). FRET-based cyclic GMP biosensors measure low cGMP concentrations in cardiomyocytes and neurons. *Commun. Biol.* 2, 1–12. doi:10.1038/s42003-019-0641-x.

Campbell, J. C., Kim, J. J., Li, K. Y., Huang, G. Y., Reger, A. S., Matsuda, S., et al. (2016). Structural basis of cyclic nucleotide selectivity in cGMP-dependent Protein Kinase II. *J. Biol. Chem.* 291, 5623–5633. doi:10.1074/jbc.M115.691303.

Chao, Y. C., Surdo, N. C., Pantano, S., and Zaccolo, M. (2019). Imaging cAMP nanodomains in the heart. *Biochem. Soc. Trans.* 47, 1383–1392. doi:10.1042/BST20190245.

Darden, T., York, D., and Pedersen, L. (1993). Particle mesh Ewald: An N·log(N) method for Ewald sums in large systems. *J. Chem. Phys.* 98, 10089–10092. doi:10.1063/1.464397.

Essmann, U., Perera, L., Berkowitz, M. L., Darden, T., Lee, H., and Pedersen, L. G. (1995). A smooth particle mesh Ewald method. *J. Chem. Phys.* 103, 8577–8593. doi:10.1063/1.470117.

Friebe, A., Sandner, P., and Schmidtko, A. (2020). cGMP: a unique 2nd messenger molecule – recent developments in cGMP research and development. *Naunyn. Schmiedebergs. Arch. Pharmacol.* 393, 287–302. doi:10.1007/s00210-019-01779-z.

Hofmann, F., Bernhard, D., Lukowski, R., and Weinmeister, P. (2009). CGMP regulated protein Kinases (cGK). *Handb. Exp. Pharmacol.* 191, 137–162. doi:10.1007/978-3-540-68964-5_8.

Honda, A., Adams, S. R., Sawyer, C. L., Lev-Ram, V., Tsien, R. Y., and Dostmann, W. R. G. (2001). Spatiotemporal dynamics of guanosine 3′,5′-cyclic monophosphate revealed by a genetically encoded, fluorescent indicator. *Proc. Natl. Acad. Sci. U. S. A.* 98, 2437–2442. doi:10.1073/pnas.051631298.

Huang, G. Y., Kim, J. J., Reger, A. S., Lorenz, R., Moon, E. W., Zhao, C., et al. (2014). Structural basis for cyclic-nucleotide selectivity and cGMP-selective activation of PKG i. *Structure* 22, 116–124. doi:10.1016/j.str.2013.09.021.

Knott, E. P., Assi, M., Rao, S. N. R., Ghosh, M., and Pearse, D. D. (2017). Phosphodiesterase inhibitors as a therapeutic approach to neuroprotection and repair. *Int. J. Mol. Sci.* 18. doi:10.3390/ijms18040696.

Lindenburg, L., and Merkx, M. (2014). Engineering genetically encoded FRET sensors. *Sensors (Switzerland)* 14, 11691–11713. doi:10.3390/s140711691.

Lissandron, V., Terrin, A., Collini, M., D’Alfonso, L., Chirico, G., Pantano, S., et al. (2005). Improvement of a FRET-based indicator for cAMP by linker design and stabilization of donor-acceptor interaction. *J. Mol. Biol.* 354, 546–555. doi:10.1016/j.jmb.2005.09.089.

Machado, M., and Pantano, S. (2015). “Structure-based, in silico approaches for the development of novel cAMP FRET reporters,” in *cAMP Signaling: Methods and Protocols* (Springer New York), 41–58. doi:10.1007/978-1-4939-2537-7_4.

Machado, M. R., Barrera, E. E., Klein, F., Sónora, M., Silva, S., and Pantano, S. (2019). The SIRAH 2.0 Force Field: Altius, Fortius, Citius. *J. Chem. Theory Comput.* 15. doi:10.1021/acs.jctc.9b00006.

Machado, M. R., and Pantano, S. (2016). SIRAH tools: Mapping, backmapping and visualization of coarse-grained models. *Bioinformatics* 32, 1568–1570. doi:10.1093/bioinformatics/btw020.

Meng, F., and Sachs, F. (2011). Visualizing dynamic cytoplasmic forces with a compliance-matched FRET sensor. *J. Cell Sci.* 124, 261–269. doi:10.1242/jcs.071928.

Niino, Y., Hotta, K., and Oka, K. (2009). Simultaneous Live Cell Imaging Using Dual FRET Sensors with a Single Excitation Light. *PLoS One* 4, e6036. doi:10.1371/journal.pone.0006036.

Nikolaev, V. O., Gambaryan, S., and Lohse, M. J. (2006). Fluorescent sensors for rapid monitoring of intracellular cGMP. *Nat. Methods* 3, 23–25. doi:10.1038/nmeth816.

Nikolaev, V. O., and Lohse, M. J. (2006). Monitoring of cAMP synthesis and degradation in living cells. *Physiology* 21, 86–92. doi:10.1152/physiol.00057.2005.

Pendin, D., Greotti, E., Lefkimmiatis, K., and Pozzan, T. (2016). Exploring cells with targeted biosensors. *J. Gen. Physiol.* 149, 1–36. doi:10.1085/jgp.201611654.

Perera, R. K., Sprenger, J. U., Steinbrecher, J. H., Hübscher, D., Lehnart, S. E., Abesser, M., et al. (2015). Microdomain switch of cGMP-regulated phosphodiesterases leads to ANP-induced augmentation of β-adrenoceptor-stimulated contractility in early cardiac hypertrophy. *Circ. Res.* 116, 1304–1311. doi:10.1161/CIRCRESAHA.116.306082.

Pettersen, E. F., Goddard, T. D., Huang, C. C., Couch, G. S., Greenblatt, D. M., Meng, E. C., et al. (2004). UCSF Chimera - A visualization system for exploratory research and analysis. *J. Comput. Chem.* 25, 1605–1612. doi:10.1002/jcc.20084.

Rosell, F. I., and Boxer, S. G. (2003). Polarized Absorption Spectra of Green Fluorescent Protein Single Crystals:  Transition Dipole Moment Directions. *Biochemistry* 42, 177–183. doi:10.1021/bi0266535.

Russwurm, M., Mullershausen, F., Friebe, A., Jäger, R., Russwurm, C., and Koesling, D. (2007). Design of fluorescence resonance energy transfer (FRET)-based cGMP indicators: A systematic approach. *Biochem. J.* 407, 69–77. doi:10.1042/BJ20070348.

Schepers, M., Tiane, A., Paes, D., Sanchez, S., Rombaut, B., Piccart, E., et al. (2019). Targeting Phosphodiesterases-Towards a Tailor-Made Approach in Multiple Sclerosis Treatment. *Front. Immunol.* 10, 1727. doi:10.3389/fimmu.2019.01727.

Sprenger, J., and Nikolaev, V. (2013). Biophysical Techniques for Detection of cAMP and cGMP in Living Cells. *Int. J. Mol. Sci.* 14, 8025–8046. doi:10.3390/ijms14048025.

Stangherlin, A., Koschinski, A., Terrin, A., Zoccarato, A., Jiang, H., Fields, L. A., et al. (2014). *Fluorescent Protein-Based Biosensors*. , eds. J. Zhang, Q. Ni, and R. H. Newman Totowa, NJ: Humana Press doi:10.1007/978-1-62703-622-1.

Stangherlin, A., and Zaccolo, M. (2012). cGMP-cAMP interplay in cardiac myocytes: A local affair with far-reaching consequences for heart function. *Biochem. Soc. Trans.* 40, 11–14. doi:10.1042/BST20110655.

Surdo, N. C., Berrera, M., Koschinski, A., Brescia, M., MacHado, M. R., Carr, C., et al. (2017). FRET biosensor uncovers cAMP nano-domains at b-adrenergic targets that dictate precise tuning of cardiac contractility. *Nat. Commun.* 8, 1–14. doi:10.1038/ncomms15031.

Vaandrager, A. B., Hogema, B. M., and De Jonge, H. R. (2005). Molecular properties and biological functions of cGMP-dependent protein kinase II. *Front. Biosci.* 10, 2150–2164. doi:10.2741/1687.

Zaccolo, M., De Giorgi, F., Cho, C. Y., Feng, L., Knapp, T., Negulescu, P. A., et al. (2000). A genetically encoded, flourescent indicator for cyclic AMP in living cells. *Nat. Cell Biol.* 2, 25–29. doi:10.1038/71345.

Zhao, D., Guallar, E., Vaidya, D., Ndumele, C. E., Ouyang, P., Post, W. S., et al. (2020). Cyclic Guanosine Monophosphate and Risk of Incident Heart Failure and Other Cardiovascular Events: the ARIC Study. *J. Am. Heart Assoc.* 9. doi:10.1161/JAHA.119.013966.
